# Opposing regulation of the late phase TNF response by mTORC1-IL-10 signaling and hypoxia in human macrophages

**DOI:** 10.1038/srep31959

**Published:** 2016-08-25

**Authors:** Linda Huynh, Anthony Kusnadi, Sung Ho Park, Koichi Murata, Kyung-Hyun Park-Min, Lionel B. Ivashkiv

**Affiliations:** 1 Graduate Program in Immunology and Microbial Pathogenesis, Weill Cornell Graduate School of Medical Sciences, 1300 York Avenue, New York, NY 10021, USA; 2 Arthritis and Tissue Degeneration Program and David Z. Rosensweig Genomics Research Center, Hospital for Special Surgery, 535 East 70th Street, New York, NY, 10021, USA.

## Abstract

Tumor necrosis factor (TNF) is best known for inducing a rapid but transient NF-κB-mediated inflammatory response. We investigated later phases of TNF signaling, after the initial transient induction of inflammatory genes has subsided, in primary human macrophages. TNF signaling induced expression of late response genes, including inhibitors of NF-κB and TLR signaling, with delayed and sustained kinetics 6–24 hr after TNF stimulation. A subset of late phase genes was expressed in rheumatoid arthritis synovial macrophages, confirming their expression under chronic inflammatory conditions *in vivo*. Expression of a subset of late phase genes was mediated by autocrine IL-10, which activated STAT3 with delayed kinetics. Hypoxia, which occurs at sites of infection or inflammation where TNF is expressed, suppressed this IL-10-STAT3 autocrine loop and expression of late phase genes. TNF-induced expression of IL-10 and downstream genes was also dependent on signaling by mTORC1, which senses the metabolic state of cells and is modulated by hypoxia. These results reveal an mTORC1-dependent IL-10-mediated late phase response to TNF by primary human macrophages, and identify suppression of IL-10 responses as a new mechanism by which hypoxia can promote inflammation. Thus, hypoxic and metabolic pathways may modulate TNF responses during chronic inflammation.

TNF-α (referred to hereafter as TNF) signals through receptors TNF-R1 and TNF-R2 and initiates a classic pro-inflammatory immune response by activating NF-κB, AP-1, and MAPK^1^. TNF signaling is necessary for optimal host defense against various pathogens^2^,^3^,^4^. Excessive TNF activity contributes to chronic inflammatory disease pathology. TNF transgenic mice, which overexpress human TNF, develop erosive arthritis similar to human rheumatoid arthritis^5^, and TNF deficiency is protective in several animal models of autoimmunity^6^,^7^. In line with this, anti-TNF therapy is used to treat autoimmune and inflammatory diseases including Crohn’s disease, rheumatoid arthritis (RA), and psoriasis^1^,^8^.

Despite our understanding of proximal and early phase TNF signaling (1–120 minutes after TNF stimulation)^1^, less is known about how longer term and chronic exposure to TNF affects cellular responses. Although the effects of chronic TNF exposure are predominantly pro-inflammatory, it is clear that TNF also has homeostatic and suppressive functions that appear to be masked or over-ridden during chronic pathological inflammation^8^. For example, early work with TNF knock out mice suggested that TNF plays a homeostatic, protective role in attenuating immune responses and allowing for tissue repair^3^. Evidence points to a protective role for TNF in various animal infectious or inflammatory disease models, for instance, TNF-R1 deficient mice infected with *Leishmania major* were capable of clearing the parasites but were unable to resolve the ensuing sterile inflammatory lesions^9^. Mechanistically, TNF can suppress production of IL-12 to decrease inflammatory damage in a mouse model of *Corynebacterium parvum* infection^10^. More recently, it has been shown that TNF can also induce cross-tolerance of macrophages to subsequent TLR stimulation, thereby suppressing excessive inflammatory cytokine production^11^. Thus, examining pathways regulated by prolonged and sustained exposure to TNF, which we refer to as the “late phase TNF response”, can provide more insight into the complex role of TNF in inflammatory responses and diseases.

Another key regulator of inflammatory and immune responses is oxygen tension, which varies *in vivo*, with tissue tension ranging from 2.5–9% O_2_ but decreasing to levels <1% O_2_ in wounds and inflammatory and necrotic tissue sites^12^. In contrast, ambient oxygen tension is approximately 20% O_2_. Oxygen demand can outstrip supply, which is termed hypoxia, during a pro-inflammatory response, when the activation, proliferation, and recruitment of cells to a confined area consumes available oxygen and diffusion is restricted. For example, hypoxia is present in the inflamed synovium of RA patients and is associated with synovial macrophage number, growth factors, and angiogenesis^13^,^14^,^15^. Oxygen-regulated pathways play important roles in the activation and antimicrobial functions of innate immune cells, including macrophages. Hypoxia-inducible transcription factors (HIFs) are stabilized under hypoxic conditions and play a key role in mediating the hypoxic response. The hypoxic response involves switching from oxidative phosphorylation to glycolysis, suppression of growth and proliferation, and induction of angiogenesis. For macrophages and neutrophils, HIFs are also important for mediating inflammatory and bactericidal responses^12^. Myeloid-specific deletion of HIF-1α or HIF-2α results in protection from LPS-mediated toxicity, decreased antibacterial responses, impairment of energy-demanding processes due to dysregulated glycolysis, and decreased disease severity in a model of collagen-induced arthritis^16^,^17^. HIF-1α can also be activated in macrophages by potent inflammatory stimuli such as TLR ligands in a hypoxia-independent manner and contributes to IL-1 production^18^. Thus, innate immune responses are tightly intertwined with hypoxic responses and pathways. However, oxygen tension is rarely taken into consideration in studies of activation mechanisms in innate immune cells.

Although HIF is the best known and extensively studied mediator of the hypoxic response, the metabolic stress of hypoxia induces additional signaling pathways that enable cells to adapt to hypoxic conditions. These include activation of the ER stress response and inhibition of mechanistic target of rapamycin complex 1 (mTORC1) signaling^19^,^20^. The mTORC1 protein complex, which contains the mTOR kinase, senses and coordinates cellular responses to the extracellular microenvironment, including growth factors, nutrients, oxygen, and stress, and mTORC1 signaling is required for anabolic, biosynthetic and proliferative pathways. Regulation of the ER stress response and mTOR signaling by oxygen tension has been studied primarily in tumor systems^19^,^20^ and has not been described in immune cell signaling. Interestingly, mTOR is emerging as a key player in immune responses^21^, typically studied in the context of its regulation by growth factors and cytokines.

mTOR is a serine/threonine kinase that signals through two distinct large, multi-protein complexes, mTORC1 and mTORC2, to regulate protein and lipid synthesis, autophagy, lysosome biogenesis, cytoskeletal organization, and metabolism^22^. mTORC1 activity in response to environmental cues is negatively regulated by the tuberous sclerosis 1 and tuberin (TSC1-TSC2) complex^23^,^24^. Many signals converge on TSC1/2 to regulate mTORC1 activity, including low oxygen. Hypoxia induces expression of REDD1 (regulated in development and DNA damage responses 1), which promotes assembly of TSC1/2 and leads to subsequent attenuation of mTORC1 activity^25^. Hypoxia also suppresses mTORC1 activity through activation of AMPK (adenosine monophosphate-activated protein kinase)^26^. AMPK increases TSC1/2 GAP activity and phosphorylates a protein in the mTORC1 complex, Raptor, to allosterically hinder mTORC1 activity^22^. In addition to being regulated by growth factors and environmental cues via the TCS1/2 pathway, mTORC1 activation is regulated by sensing of intracellular nutrients such as amino acids, which induces translocation of the complex to lysosomal membranes where it is activated^22^,^27^.

mTORC1 regulates innate immune functions in part by regulating translation^22^,^28^,^29^ and cytokine production, including type I interferon production in plasmacytoid dendritic cells^30^. In macrophages and lamina propria DCs, mTORC1 activity is required for optimal IL-10 induction by LPS, and inhibition of mTORC1 increases expression of pro-inflammatory cytokines such as IL-12^31^,^32^. mTORC1 promotes cytokine production and chemotaxis in mast cells^33^ and neutrophils^34^ and augments extracellular trap formation^35^. Overall, mTORC1 has counterbalancing activating and suppressive functions in innate immune cells and thus the functional outcomes of mTORC1 signaling are context dependent.

We are interested in the later phases of macrophage responses to TNF, which occur after the initial transient induction of canonical inflammatory genes such as IL-6 and IL-8 has subsided. Previous work in our laboratory had shown that TNF induces a late phase interferon (IFN) response (apparent 1–2 days after TNF stimulation) that is mediated by autocrine IFN-β^36^, followed by an NFATc1-mediated program of differentiation into osteoclast-like cells (apparent 3–6 days after TNF stimulation)^37^. In this study, we found that TNF induces a delayed IL-10-mediated autocrine loop characterized by secretion of IL-10 and activation of STAT3 that became apparent 1–2 days after TNF stimulation. Autocrine IL-10 contributes to late phase TNF-induced expression of genes that are not induced during the early, acute pro-inflammatory phase of TNF signaling and were not previously known as TNF target genes. These late phase TNF genes include several negative regulators of inflammatory responses and NF-κB signaling, including *TNIP3* (encodes ABIN-3, which interacts with A20), *CLU* (encodes clusterin), and *SIGLEC10*, suggesting a homeostatic component to this late response. This late phase gene induction was partially dependent on mTORC1 and was abrogated by hypoxia. These results identify a new component of the TNF response and demonstrate its regulation by hypoxia. The results highlight a role for TNF-mediated feedback inhibition and the importance of metabolic and stress pathways in cross-regulating immune signaling pathways.

## Results

### Induction of delayed and sustained gene expression by TNF

**Figure 1:**
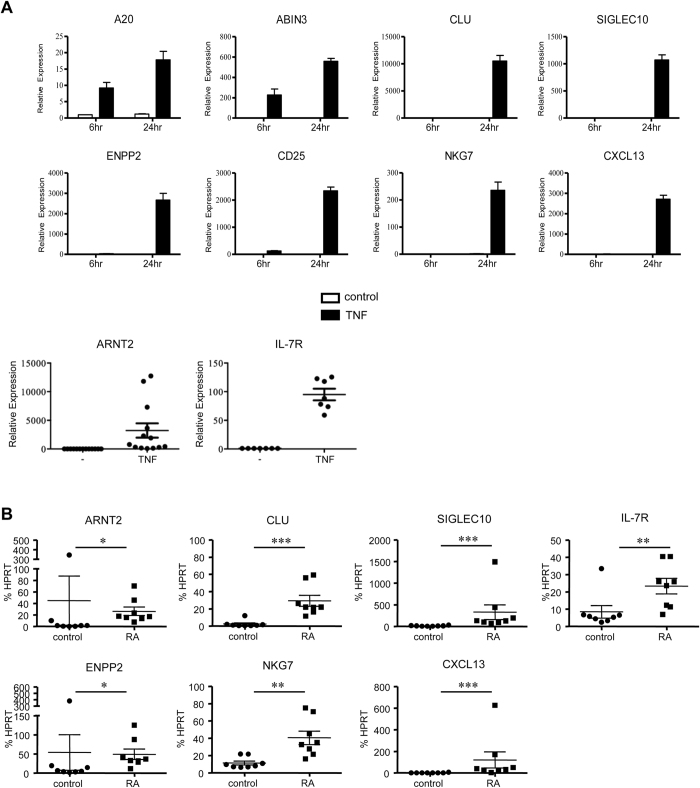
TNF signaling induces late-phase response genes. (**A**) Primary human macrophages were stimulated with TNF (10 ng/mL, black bars) for 6 or 24 hours. mRNA expression was measured by qPCR and results are presented as mean ± SD of duplicate wells normalized relative to GAPDH. Data are representative of at least 3 independent experiments. For ARNT2 and IL7R, data from individual donors at the 24 hour time point is shown. Each symbol represents a different donor, and the cumulative mean ± SEM for all donors is shown. (**B**) Late-phase TNF inducible genes are expressed in RA synovial macrophages. mRNA expression in freshly isolated RA synovial macrophages and control blood-derived macrophages was measured by qPCR and results are presented as mean ± SEM normalized relative to HPRT. Statistical analysis was performed using the Mann-Whitney test. n = 8 for both groups. *P ≤ 0.05; **P ≤ 0.01; ***P ≤ 0.001.

In previous work we found that activation of human macrophages with various factors, including TNF, activated a subset of genes whose expression was not apparent until 6–24 hours after stimulation and was sustained for several days^11^,^36^,^37^,^38^,^39^. In microarray experiments analyzing the kinetics of gene expression during macrophage activation, we identified genes that are highly induced at the 24 hour time point (Supplementary Table SI and SII). Strikingly, expression of the majority of these genes peaked 6 or 24 hours after stimulation, often with minimal induction at the 1 and 3 hour time points (Supplementary Table SI). Genes that exhibit a delayed and sustained pattern of gene expression are hereafter termed late phase genes. We confirmed late phase expression of several of these genes after TNF stimulation by quantitative real time PCR (qPCR) (Fig. 1A). In this study we focused on genes that have known inhibitory functions (*A20, ABIN3, CLU, SIGLEC10*) and genes strongly induced with markedly delayed kinetics (*ENPP2* which encodes autotaxin, *CD25, NKG7, CXCL13, ARNT2* and *IL7R*) (Fig. 1A). These genes were also selected for study because they were highly expressed in synovial macrophages from rheumatoid arthritis (RA) patients that are chronically exposed to TNF *in vivo* (Fig. 1B), which confirms their expression under chronic inflammatory conditions *in vivo* and supports their potential (patho)physiological importance. The data indicate that TNF signaling induces late phase expression of genes that are expressed during TNF-driven inflammation in RA, but are not expressed during the early and transient classic pro-inflammatory TNF response that occurs 1–3 hours after TNF stimulation.

### TNF induces production of IL-10 and activation of STAT3

**Figure 2:**
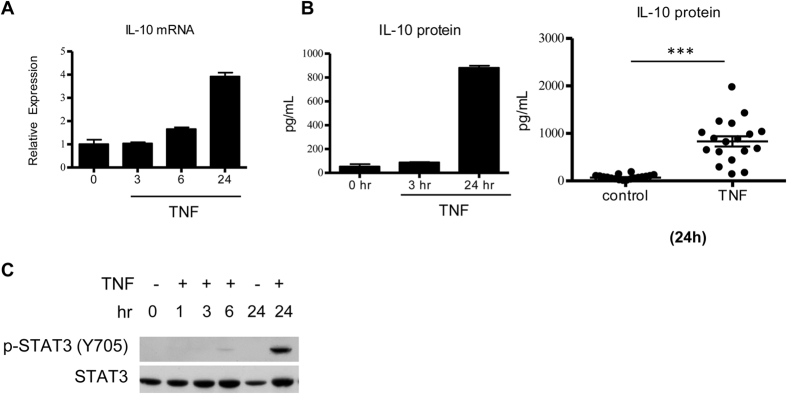
TNF signaling induces late-phase IL-10 production and STAT3 activation. Primary human macrophages were stimulated with TNF (10 ng/mL) for the indicated times. (**A**) TNF signaling induces delayed expression of IL-10 mRNA. mRNA expression was measured by qPCR, and results are presented as mean ± SD of duplicate wells normalized relative to HPRT. Data are representative of at least 3 independent experiments. (**B**) TNF signaling induces delayed production of IL-10 protein. IL-10 protein from culture supernatants was measured by ELISA. Data from one donor is shown in the bar graph, and data from 18 individual donors is shown on the right. Each symbol represents a different donor, and the cumulative mean ± SEM for all donors is shown. Data was analyzed using the Wilcoxon matched-pairs signed-rank test. ***P = 0.0003. (**C**) TNF signaling induces delayed activation of STAT3. Whole cell lysates were immunoblotted with Abs against phospho-STAT3 (Y705) and STAT3. Data are representative of 3 independent experiments.

We noted that several of the late phase TNF response genes, namely *ENPP2, IL7R* and *ARNT2*, correspond to genes previously identified by our laboratory as IL-10 inducible in primary human macrophages^40^,^41^. IL-10 is well known to be rapidly and transiently induced by TLR ligands and mediates a key feedback inhibitory loop^42^,^43^. Induction of IL-10 by TNF was described in 1993^44^ but to our knowledge this finding was not followed up, and regulation of TNF-induced autocrine IL-10 production and downstream signaling, gene induction and function have not been investigated. We investigated induction of IL-10 in our system, and whether IL-10 contributes to expression of late phase TNF response genes. In contrast to early induction of IL-10 by TLRs, which is observed 1–3 hours after stimulation, induction of IL-10 mRNA by TNF was delayed, as it was minimally increased 6 hours and further increased 24 hours after TNF stimulation (Fig. 2A). Accordingly, substantial amounts of secreted IL-10 protein were detected in culture supernatants 24 hours after TNF stimulation, reaching concentrations of >1 ng/ml in many donors (Fig. 2B). Furthermore, IL-10 mRNA and protein amounts continued to increase for up to 48 hours after TNF stimulation (data not shown). These concentrations of IL-10 are sufficient to activate STAT3 and gene expression in human macrophages, and indeed we observed induction of STAT3 tyrosine phosphorylation 24 hours after TNF stimulation (Fig. 2C and Supplementary Fig. S1), which was substantially delayed relative to activation of STAT3 that was readily detectable 3 hours after LPS stimulation (Supplementary Fig. S1). These results show that TNF induces delayed expression of IL-10 and suggest that this IL-10 may activate signals to modulate the late phase TNF response.

**Figure 3:**
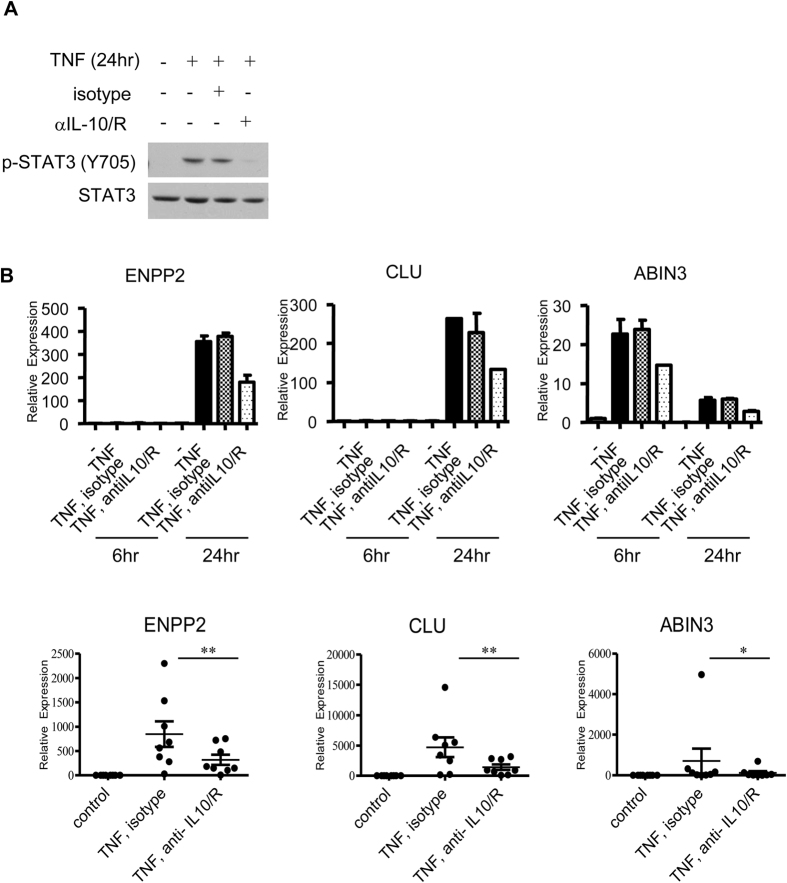
TNF-induced STAT3 activation and specific gene induction are IL-10 dependent. Primary human macrophages were pre-incubated with IL-10 neutralizing and IL-10R blocking antibodies (5 μg/mL) and then stimulated with TNF (10 ng/mL) for the indicated times. Isotype controls for both neutralizing and blocking antibodies (5 μg/ml) were used. Polymyxin B was included in all conditions to neutralize potential endotoxin contamination from the antibody stock solutions. (**A**) TNF-induced STAT3 activation is IL-10 dependent. Whole cell lysates were immunoblotted with Abs against phospho-STAT3 (Y705) and STAT3. Data are representative of at least 3 independent experiments. (**B**) mRNA expression was measured by qPCR, and results from one donor are presented as mean ± SD of duplicate wells normalized relative to HPRT (top graphs). Bottom graphs show data from 8 individual donors. Each symbol represents a different donor, and the cumulative mean ± SEM for all donors is shown. Data was analyzed using the Wilcoxon matched-pairs signed-rank test (bottom graphs). *P ≤ 0.05; **P ≤ 0.01.

We wished to test whether STAT3 activation downstream of TNF was predominantly mediated by autocrine IL-10, as STAT3 can be activated by other TNF-inducible factors such as IL-6, leptin, and VEGF^45^. We found that blocking IL-10 using neutralizing antibodies and IL-10R blocking antibodies essentially completely suppressed TNF-induced STAT3 activation (Fig. 3A). Thus, autocrine IL-10 is the predominant STAT3-activating factor induced by TNF. IL-10 blockade also partially but significantly suppressed TNF-induced expression of a subset of late phase genes, namely *ENPP2* (P < 0.01), *CLU* (P < 0.01) and *TNIP3* (P < 0.05) (Fig. 3B); expression of *SIGLEC10* and *ARNT2* was also attenuated but the decrease was not statistically significant (data not shown). The partial suppression of gene expression may be secondary to incomplete blockade of IL-10 or may reflect activation by distinct IL-10-independent pathways. These results show that TNF induces an IL-10-mediated delayed autocrine loop that activates STAT3 and contributes to late phase gene expression. As clusterin (encoded by *CLU*) and ABIN-3 (encoded by *TNIP3*) are negative regulators of NF-κB and inflammatory signaling^46^,^47^, the results suggest a suppressive component to TNF-induced IL-10 that functions at later stages of macrophage activation, which would parallel the suppressive function of TLR-induced IL-10 that functions at earlier stages of macrophage activation.

### Hypoxia selectively inhibits TNF-induced, IL-10-dependent responses

**Figure 4:**
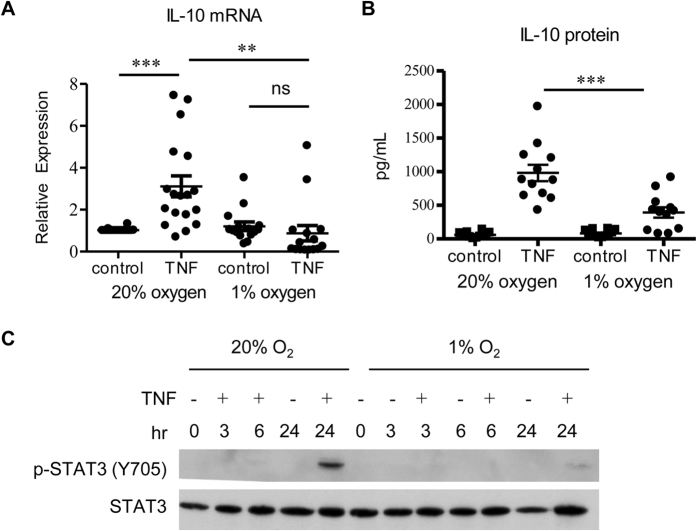
Hypoxia inhibits TNF induced IL-10 production and STAT3 activation. Primary human macrophages were differentiated with M-CSF at normoxia (20% O_2_) and then moved into 1% O_2_ for 3 h before TNF (10 ng/mL) was added. Control cells were maintained at normoxia. (**A**) Hypoxia inhibits TNF mediated IL-10 mRNA expression. Cells were stimulated with TNF for 24 h. mRNA expression was measured by qPCR and normalized relative to HPRT. Data from 15 individual donors is shown. Each symbol represents a different donor, and the cumulative mean ± SEM for all donors is shown. Data was analyzed using the Wilcoxon matched-pairs signed-rank test. **P ≤ 0.01; ***P ≤ 0.001; ns:not significant. (**B**) IL-10 protein from culture supernatants was measured by ELISA. Data from 12 individual donors and the cumulative mean ± SEM are shown. Data was analyzed using the Wilcoxon matched-pairs signed-rank test. ***P = 0.0005 (**C**) Hypoxia inhibits STAT3 activation. Whole cell lysates were immunoblotted with Abs against phospho-STAT3 (Y705) and STAT3. Data are representative of at least 3 independent experiments.

TNF signaling and late phase responses during chronic inflammation, such as in RA synovium, occur in the context of a hypoxic microenvironment^13^,^14^,^15^. Hypoxia is generally considered to be pro-inflammatory and thus we tested whether hypoxia could suppress TNF-induced IL-10 production and downstream gene expression. We first established that exposure of primary human macrophages to hypoxia (1% O_2_, reflects inflammatory sites) strongly induced canonical hypoxia response HIF-1α target genes *GLUT1, VEGF* and *GAPDH* relative to normoxia (20% O_2_) or physiological tissue oxygen tension (10% O_2_) (Supplementary Fig. S2A). Accordingly, hypoxia induced expression of HIF-1α protein (Supplementary Fig. S2B); levels of hypoxia-induced HIF-1α protein were not consistently modulated by TNF in different donors. We then tested whether TNF-induced IL-10 and late phase gene expression were affected by hypoxia. Interestingly, hypoxia suppressed TNF-induced IL-10 mRNA expression in multiple donors (Fig. 4A). Likewise, induction of IL-10 protein was also suppressed by hypoxia (Fig. 4B), as was activation of STAT3 (Fig. 4C).

**Figure 5:**
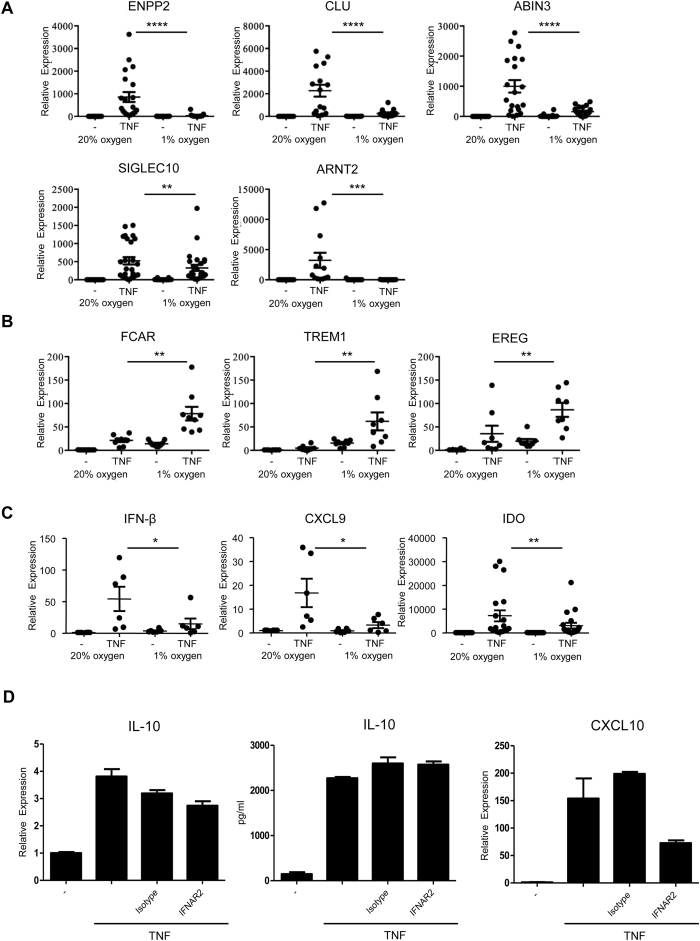
Hypoxia inhibits a subset of late-phase TNF inducible genes. Primary human macrophages were differentiated with M-CSF at normoxia (20% O_2_) and then moved into 1% O_2_ for 3 h before TNF (10 ng/mL) was added for an additional 24 h. Control cells were maintained at normoxia. mRNA expression was measured by qPCR and normalized relative to HPRT. (**A**) Hypoxia inhibits select TNF inducible genes, including IL-10 dependent genes. Data from at least 14 independent donors was analyzed using the Wilcoxon matched-pairs signed-rank test. **P ≤ 0.01; ***P ≤ 0.001; ****P ≤ 0.0001. (**B**) Hypoxia augments TNF-induced expression of select genes, including genes with inflammatory functions. Data from at least 9 independent donors was analyzed using the Wilcoxon matched-pairs signed-rank test. *P ≤ 0.05; **P ≤ 0.01. (**C**) Hypoxia inhibits TNF-induced expression of IFN-β and interferon-stimulated genes. Data from at least 6 independent donors was analyzed using the Wilcoxon matched-pairs signed-rank test. *P ≤ 0.05; **P ≤ 0.01. (**D**) Autocrine type I IFNs have minimal effect on TNF-induced IL-10 production. Blocking antibodies against the type I IFN receptor (1 μg/ml) were added at the initiation of cultures. Representative results from three experiments are shown.

Next, we examined expression of the late-phase TNF inducible genes that were analyzed in Fig. 1. Of these, the genes that were clearly dependent on IL-10 (Fig. 3B), *ENPP2, CLU*, and *TNIP3*, were strongly suppressed by hypoxia (Fig. 5A). In addition *SIGLEC10* and *ARNT2*, whose expression trended down when IL-10 was blocked, were suppressed by hypoxia (Fig. 5A), while other late phase genes including *CD25* were not suppressed (Supplementary Fig. S3A and data not shown). Suppression of ABIN3 (encoded by *TNIP3*) and ARNT2 by hypoxia at the protein level was confirmed using immunoblotting (Supplementary Fig. S4). As an additional specificity control, we found that the late phase TNF response genes *FCAR, TREM1, EREG*, which have inflammatory functions, were super-induced by hypoxia (Fig. 5B). Our lab has previously shown that TNF can induce expression of IFN-β and type I IFN response genes^36^. Hypoxia suppressed TNF-induced expression of *IFNB* and downstream interferon response genes, including *CXCL9* and *IDO* (Fig. 5C). IFN-β has been previously shown in murine macrophages to augment TLR-induced IL-10 expression^48^, and thus we addressed whether decreased IFN-β production could contribute to lower IL-10 expression in our human system. However, blockade of the IFN receptor had only a modest effect on TNF-induced IL-10 mRNA expression and did not result in decreased IL-10 protein production (Fig. 5D), suggesting that downregulation of IL-10 by hypoxia does not occur solely secondary to diminished IFN-β levels. Overall, the results show that hypoxia suppresses late phase TNF-induced gene expression, including suppression of IL-10- and IFN-β-mediated autocrine loops and their respective downstream target genes.

**Figure 6:**
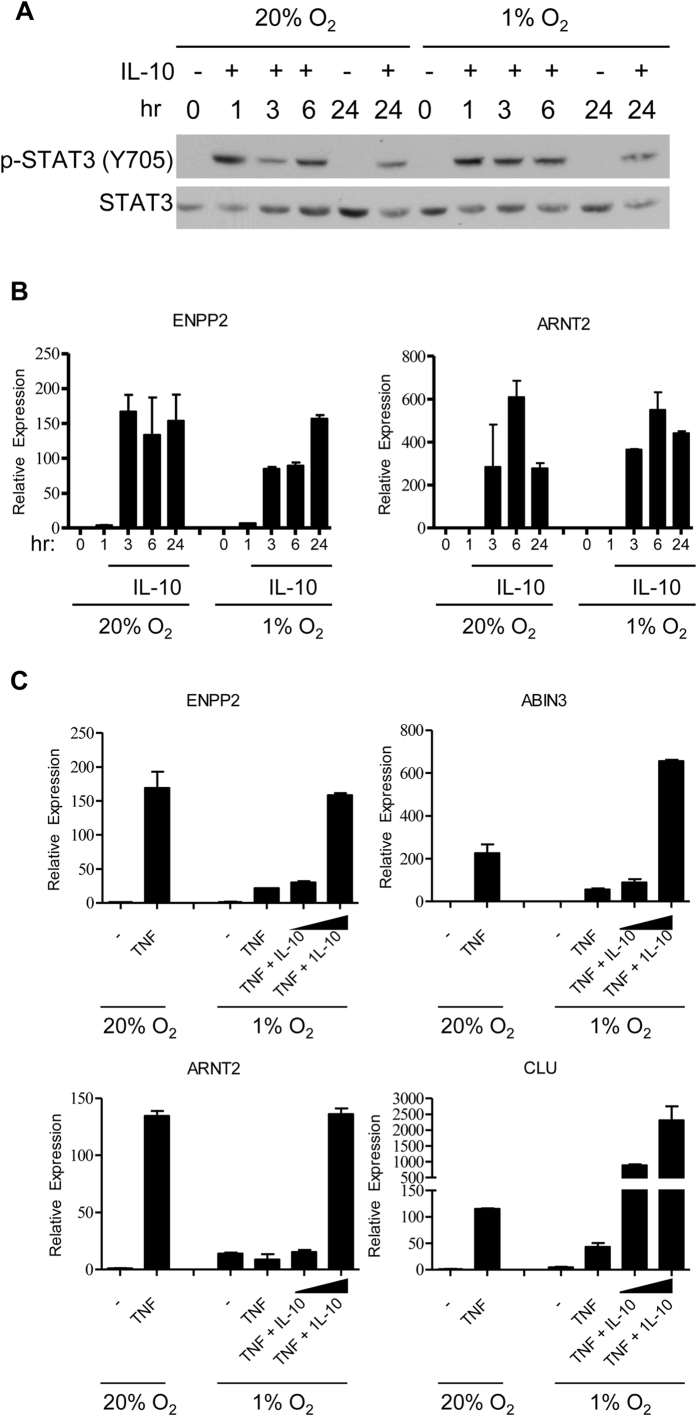
Hypoxia does not inhibit IL-10 signaling in primary human macrophages. (**A,B**) Primary human macrophages were differentiated with M-CSF at normoxia (20% O_2_) and then moved into 1% O_2_ for 3 h before IL-10 (10 ng/mL) was added for the indicated times. Control cells were maintained at normoxia. (**A**) Hypoxia does not inhibit STAT3 activation downstream of exogenous IL-10. Whole cell lysates were immunoblotted with Abs against phospho-STAT3 (Y705) and STAT3. Data are representative of 3 independent experiments. (**B**) Hypoxia does not inhibit induction of IL-10 response genes. mRNA expression was measured by qPCR, and results are presented as mean ± SD of duplicate wells normalized relative to HPRT. Data are representative of at least 3 independent experiments. (**C**) Exogenous IL-10 restores expression of TNF-inducible, hypoxia-suppressed genes. Primary human macrophages were differentiated with M-CSF at normoxia (20% O_2_) and then moved into 1% O_2_ for 3 h before TNF (10 ng/mL) was added. IL-10 (10 or 100 ng/mL) was added 6 h after TNF. Control cells were maintained at normoxia. mRNA expression was measured by qPCR, and results are presented as mean ± SD of duplicate wells normalized relative to HPRT. Data are representative of two independent experiments.

It remained possible that hypoxia suppressed IL-10 signaling in addition to suppressing IL-10 production. We tested this possibility by analyzing the effects of hypoxia on signaling and gene induction in response to exogenous IL-10. However, IL-10-induced STAT3 activation and gene induction were not suppressed by hypoxia (Fig. 6A,B). In addition, we found that adding back exogenous IL-10 to hypoxic TNF-treated macrophages restored expression of *ENPP2, TNIP3, ARNT2* and *CLU* to, at a minimum, levels induced by TNF under normoxic conditions (Fig. 6C). Together, these results show that hypoxia does not suppress IL-10 signaling and suggest that inhibition of IL-10 production is an important mechanism by which hypoxia suppresses TNF-mediated induction of IL-10-dependent late phase genes.

### TNF-induced, IL-10-mediated responses are dependent on mTORC1

**Figure 7:**
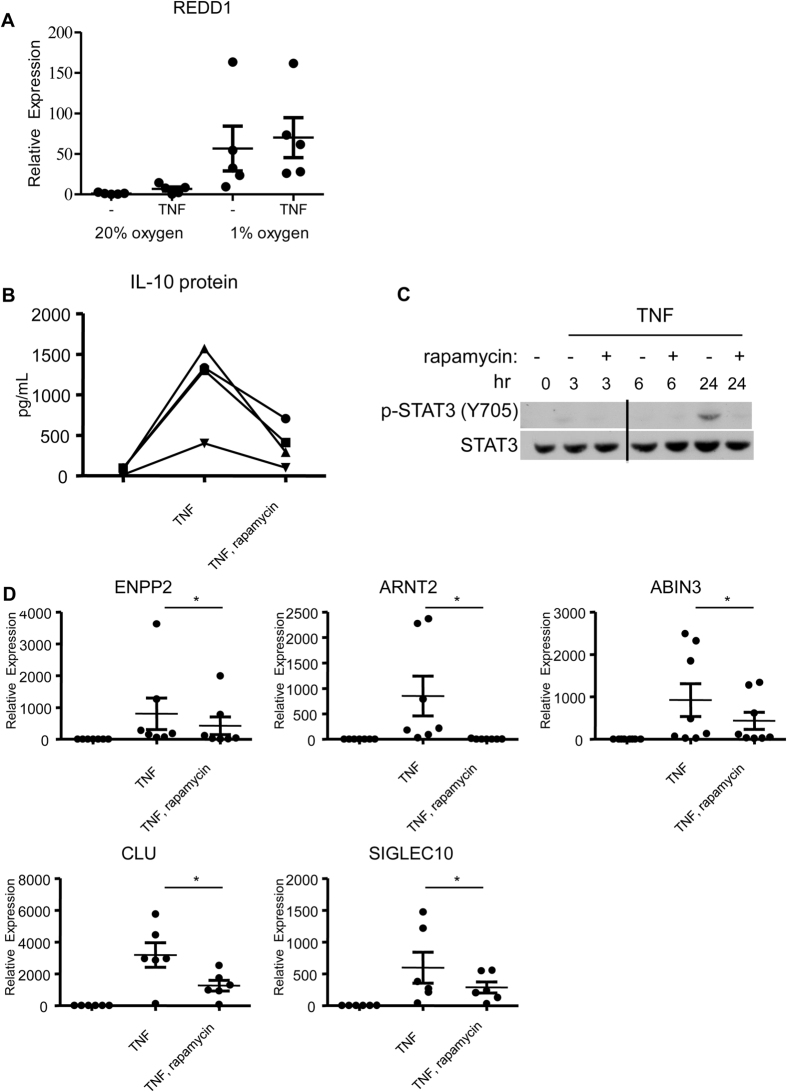
mTOR signaling is required for TNF-induced expression of IL-10 and downstream target genes. Primary human macrophages were differentiated with M-CSF at normoxia (20% O_2_) and then moved into 1% O_2_ for 3 h before TNF (10 ng/mL) was added for an additional 24 h. Control cells were maintained at normoxia. (**A**) REDD1, a negative regulator of mTOR signaling, is induced by hypoxia. mRNA expression was measured by qPCR and normalized relative to HPRT. Data from 5 individual donors are presented as mean ± SEM. (**B–D**) Primary human macrophages were pre-incubated for 1 hour with vehicle control DMSO or the mTOR inhibitor rapamycin and then stimulated with TNF (10 ng/mL) for the indicated times. These experiments were performed at normoxia. (**B**) TNF-induced IL-10 production is mTOR dependent. IL-10 protein from culture supernatants was measured by ELISA. Cumulative data from 4 independent donors are presented. (**C**) TNF-induced STAT3 activation is mTOR dependent. Whole cell lysates were immunoblotted with Abs against phospho-STAT3 (Y705) and STAT3. Data are representative of 3 independent experiments. Two parts of a blot from the same experiment have been spliced together (marked by a vertical black line between lanes 3 and 4). (**D**) TNF-induced, IL-10-dependent genes are mTOR dependent. mRNA expression was measured by qPCR and normalized relative to HPRT. Data from at least 6 individual donors is presented as mean ± SEM and was analyzed using the Wilcoxon matched-pairs signed-rank test. *P ≤ 0.05.

We wished to identify the pathway by which hypoxia suppressed IL-10 production. We reasoned that one of the three major hypoxia-regulated pathways previously described in other cell types, namely induction of HIFs, induction of the ER stress response, or suppression of mTORC1 signaling, could impact IL-10 expression. We attempted RNAi-mediated knock down of HIF expression but found that several specific and scrambled control siRNAs nonspecifically blocked the TNF-induced IL-10 response; thus the role of HIFs could not be assessed in our primary human macrophage system. Activation of the ER stress response, as assessed by alternative splicing of hXBP1 or expression of ATF4^20^,^49^ was not detected (data not shown). We then turned our attention to the mTORC1 pathway, which is suppressed at low oxygen tensions^22^ and is required for optimal IL-10 induction and translation in other systems^30^,^31^,^50^,^51^. We hypothesized that mTORC1 activity is required for TNF-induced IL-10 and suppression of mTORC1 activity by hypoxia may explain inhibition of IL-10 expression. Consistent with our recent report^29^, we detected basal mTORC1 activity in primary human macrophages. As expected^25^,^52^,^53^, hypoxia induced expression of REDD1, an inhibitor of mTORC1 (Fig. 7A). Although attempts to directly measure regulation of mTORC1 activity by TNF and hypoxia were not conclusive because of donor variability, the REDD1 data in the context of the published literature^25^,^51^,^52^ raised the possibility that mTORC1 activity is required for TNF-induced expression of IL-10 and downstream late phase genes. In support of this notion, the highly selective mTORC1 inhibitor rapamycin suppressed TNF-induced IL-10 protein production (Fig. 7B), downstream STAT3 activation (Fig. 7C) and induction of IL-10-dependent genes (Fig. 7D). As a specificity control, late-phase TNF inducible genes that were unaffected by low oxygen were also unaffected by rapamycin (Supplementary Fig. S3B). Overall, the data indicate that the late phase TNF-induced IL-10 response is dependent on mTORC1, and suggest that hypoxia may suppress this response in part by suppressing mTORC1 activity.

## Discussion

Recent work, including from our laboratory, has extended our understanding of cellular responses to TNF by examining later phases of the TNF response that differ from the extensively studied early and transient inflammatory response. In this study, we have uncovered a new aspect of the TNF response in human macrophages, namely delayed induction of an IL-10-mediated autocrine loop that activates STAT3 and downstream genes, some of which are inhibitors of inflammatory signaling and NF-κB activation. Interestingly, induction of the IL-10-STAT3 loop was dependent on mTORC1, which senses the metabolic state of the cell, thus providing a connection between macrophage metabolism and the anti-inflammatory IL-10-STAT3 pathway. TNF-mediated induction of the IL-10-STAT3 autocrine loop was abrogated by the cellular stress induced by hypoxia, suggesting a new way by which hypoxia can promote inflammation. Our results provide insight into how stress/hypoxia, metabolic, and inflammatory signaling pathways are integrated to determine late phase macrophage responses to TNF that are likely important in chronic inflammatory settings.

Autocrine loops that feed back to regulate macrophage or DC phenotype have been well described in cell responses to TLR stimulation. TLR stimulation induces at least two important autocrine feed back loops that become rapidly operational, within 1–3 hours after stimulation. One is mediated by IFN-β, which induces expression of interferon-stimulated genes (ISGs), including chemokines and molecules important for antigen presentation that are overall pro-inflammatory. At the same time, TLRs induce an IL-10-STAT3-mediated autocrine loop that serves as a potent feed back inhibitor of the early inflammatory phase of TLR-induced macrophage activation^54^. A key function of IL-10-STAT3 signaling is to limit inflammatory activation of macrophages by repressing transcription of cytokine genes such as *TNF* and *IL6*; in contrast, IL-10 does not consistently suppress upstream TLR signaling. STAT3 does not directly repress transcription of cytokine genes; instead, it has been proposed that STAT3 acts indirectly by inducing transcriptional repressors that can bind to cytokine gene promoters and enhancers^53^,^55^,^56^. There is crossregulation between TLR-induced IFN-β− and IL-10-mediated responses, as IFN-β can augment IL-10 expression^57^, while IL-10 has the potential to modulate IFN signaling via induction of SOCS3. Our work shows that, in addition to TLRs, TNF also induces both IFN-β- and IL-10-mediated feed back loops, but with much slower kinetics than induction by TLR ligands; expression of IFN-β and IL-10 target genes did not peak until 6–24 hours after TNF stimulation. Similar to TLR-induced IFN-β, TNF-induced IFN-β induces expression of chemokines and interferon stimulated genes (ISGs) and appears to have a predominantly inflammatory function^36^. Interestingly, in the context of a late phase TNF response, IL-10 contributes to expression of genes that attenuate TLR signaling (*SIGLEC10*)^58^ or suppress NF-κB activation (*CLU, TNIP3*)^47^,^59^. This suggests that the anti-inflammatory mechanisms of IL-10 in the early phase of an LPS response *versus* the late phase of a TNF response are distinct, and mediated by, respectively, transcriptional repression and inhibition of signaling. This inhibition of inflammatory signaling may contribute to the ‘tolerized’ state induced by prolonged TNF stimulation, in which inflammatory signaling in response to challenge by TLR ligands is attenuated^11^. As the TNF-induced IL-10 response peaks later and is more sustained than the TNF-induced IFN response, it is possible that TNF-induced IL-10 contributes to the attenuation of IFN responses at later time points after TNF stimulation^37^.

Metabolic pathways regulate cell fate and function of immune cells. For instance, HIF-1α and mTOR mediated glycolysis is an important checkpoint for the differentiation of T_H_17 and T_reg_ cells, with glycolysis necessary for development of T_H_17 cells and inhibition of glycolysis skewing towards T_reg_ development^60^. Similarly, glycolysis rates are higher in M1 macrophages classically activated by IFN-γ and LPS than in M2 macrophages alternatively activated by IL-4, which have higher rates of oxidative phosphorylation^61^. A body of literature (reviewed in ref. 62) suggests that the kinase AMPK, which senses cellular energy depletion and concomitantly suppresses anabolic pathways and promotes oxidative phosphorylation, also serves anti-inflammatory functions and promotes M2 polarization. We provide another link between metabolism and inflammatory responses by showing that mTORC1, which senses signaling by growth factors and requires an abundant supply of intracellular nutrients such as amino acids for its activity, also plays a role in anti-inflammatory responses by enabling late phase IL-10 production in response to TNF. mTORC1 activity was required for induction of IL-10, and a subset of late phase TNF genes that are IL-10-dependent were exquisitely sensitive to the mTORC1 inhibitor rapamycin. As AMPK and mTOR crossregulate each other’s activity, it appears that the integrated metabolic status of macrophages that is sensed by various downstream kinases plays an important role in determining the balance between pro- and anti-inflammatory responses to environmental stimuli.

The important role of hypoxia-regulated pathways and factors, like the HIFs, in innate immune cell function is well established^12^. Previous work has implicated HIFs in modulating the early phase of inflammatory macrophage activation, with mechanisms related to promoting glycolysis and maintaining cellular energy stores and augmenting IL-1 production^18^,^60^,^61^. We show that hypoxia can also augment inflammatory responses by the distinct mechanism of suppression of production of anti-inflammatory IL-10, thus abrogating late phase feed back inhibition of TNF signaling. This is reminiscent of one mechanism of pro-inflammatory IFN-γ action, which augments early TLR responses by inhibiting IL-10 expression, and supports the idea that abrogation of negative feedback loops is an important component of augmentation of innate immune responses^63^. HIFs are the best-established mediators of the effects of hypoxia on immune responses, and it is possible that HIFs contribute to hypoxia-mediated suppression of the late phase TNF-induced IL-10 response. However, we were not able to address this directly in our primary human macrophage system, as various siRNAs including scrambled controls suppressed the late phase TNF-induced IL-10 response.

The inflamed synovium of RA patients is characterized by chronic exposure to TNF and also by hypoxia^13^,^14^,^15^. Expression of the late phase TNF response genes identified in this study in RA synovial macrophages supports that these pathways and genes are expressed in chronic inflammatory settings such as RA, where they could be regulated by hypoxia and the signaling pathways we have described. RA macrophages were obtained from synovial fluids from joints that were aspirated for clinical indications, and thus it is likely that in many samples disease was locally active in the aspirated joint. The importance of synovial hypoxia in arthritis pathogenesis is becoming increasingly appreciated, and has been linked to increased production of inflammatory mediators, cytokines, chemokines, and angiogenic factors^64^. Our findings identifying late phase TNF response genes and showing their regulation by hypoxia sets the stage for future work to test the relationship of gene expression to disease activity, whether these genes are expressed in blood monocytes and macrophages from RA patients, and to test the response of RA macrophages to *ex vivo* TNF exposure, variations in oxygen tension, and rapamycin. It is possible that responses could be different between healthy and RA macrophages, and these differences could yield insights into disease pathogenesis.

This study highlights the importance of incorporating the role of low oxygen tension, which is rarely done in mechanistic *in vitro* studies because of practical limitations, into investigation of inflammatory signaling pathways. In addition to our work, it was recently shown that differentiating monocyte-derived, primary human macrophages under 5–10% O_2_, among other factors, resulted in cells that better mimicked tissue macrophages in the context of *Mycobacterium tuberculosis* infection^65^. Hypoxia is clearly important in chronic inflammatory lesions such as rheumatoid synovitis, and more accurately mimicking these hypoxic conditions in mechanistic studies with primary human cells will allow us to better understand and therapeutically manipulate chronic inflammatory responses.

In summary, TNF induces a late phase response characterized by mTORC1-dependent induction of an IL-10-STAT3-mediated autocrine loop and a late phase gene response that differs markedly from the early inflammatory gene response. Late-phase TNF signaling is regulated by environmental and metabolic pathways, including those activated by hypoxia, that are sensed and integrated by mTOR. In macrophages, hypoxia overrides the induction of feedback signaling in response to TNF and thereby alters the balance between activating and suppressive TNF signaling that determines the potency of TNF as a promoter of inflammation.

## Methods

### Cell culture and reagents

Peripheral blood mononuclear cells (PBMC) obtained from blood leukocyte preparations purchased from the New York Blood Center were separated by density gradient centrifugation with Ficoll (Invitrogen, Carlsbad, CA, USA) using a protocol approved by the Hospital for Special Surgery Institutional Review Board that adheres to NIH guidelines and regulations. Human monocytes were purified from PBMCs immediately after isolation by positive selection with anti-CD14 magnetic beads, as recommended by the manufacturer and previously described^66^ (Miltenyi Biotec, Auburn, CA, USA). The purity of isolated monocytes and expression of macrophage markers was assessed by flow cytometry using a FACS Canto (BD Biosciences) and the results are shown in Supplementary Figs S5 and S6. The antibodies used for flow cytometry were purchased from Biolegend (CD3-PB clone HIT3a (300329), CD19-PE/Cy7 clone HIB19 (302215), CD14-Alexa 647 clone HCD14 (325611), CD11b-FITC clone ICRF44 (301329), CD64-BV421 clone 10.1 (305019) and CD68-APC/Cy7 clone Y1/82A (333821)). Monocytes were cultured in RPMI 1640 medium (Invitrogen) supplemented with defined FBS (Hyclone), penicillin/streptomycin (Invitrogen), L-glutamine (Invitrogen), and 15 ng/mL human macrophage colony-stimulating factor (M-CSF; Peprotech). Cells were plated at a density of 2 million per mL, and monocyte-derived macrophages were obtained after 1–2 days of culture with human M-CSF. Synovial fluids from RA patients were obtained by their physicians as a part of standard medical care using a protocol approved by the Institutional Review Board of the Hospital for Special Surgery that adheres to NIH guidelines and regulations. De-identified specimens that would otherwise have been discarded were used in this study and were obtained under a waiver of consent. Mononuclear cells were isolated from synovial fluids by density-gradient centrifugation with Ficoll, and CD14+ cells were purified using anti-CD14 magnetic beads and characterized as previously described^67^,^68^. RA cells were not cultured but lysed directly for RNA isolation. For hypoxia experiments, performed in the laboratory of Dr. Carl Nathan (Weill Cornell Medical College), cells were maintained at 1% or 10% O_2_ and 5% CO_2_ at 37 °C in a chamber flushed with N_2_ under the control of a PRoOX sensor and ProCO_2_ regulator (BioSpherix). Cells were incubated in the 1% oxygen chamber for 3 hours prior to TNF or IL-10 stimulation, as 3 hours was sufficient to detect HIF1α protein stabilization in our system. Recombinant human TNF-α (10 ng/mL) and IL-10 (10–100 ng/mL) were purchased from Peprotech. Rapamycin (10 nM, 1 uM) and desferoxamine (200 uM) were purchased from EMD Millipore, Billerica, MA, USA. Polymyxin B (14 ug/mL) was purchased from Sigma Aldrich, St. Louis, MO, USA. IL-10 and IL-10R blocking antibodies were purchased from R&D Systems, Minneapolis, MN, USA, and used at 5 ug/mL.

### Immunoblot analysis

For immunoblotting, whole cell lysates were fractionated on 7.5% or 10% polyacrylamide gels using SDS-PAGE and transferred to polyvinylidene difluoride membranes for probing with Ab. ECL was used for detection. The following Abs were used: pY-STAT3 (Y705) (Cell Signaling Technology, Danvers, MA, USA), STAT3 (Cell Signaling), HIF-1 alpha (Cell Signaling), p38α (C-20) (Santa Cruz Biotechnology, Santa Cruz, CA, USA), and beta-tubulin (Abcam, Cambridge, MA, USA).

### ELISA

Sandwich ELISA using paired human IL-10 Abs was performed according to the manufacturer’s instructions (BD Pharmingen, San Diego, CA, USA).

### mRNA isolation and real-time quantitative PCR (qPCR)

RNA was extracted using the RNeasy Mini Kit (Qiagen, Valencia, CA, USA) and reverse-transcribed using the RevertAid First Strand cDNA Synthesis Kit (Fermentas, Glen Burnie, MD, USA) following the manufacturer’s instructions. Quantitative real-time PCR was performed in duplicate using Fast SYBR Green Master Mix and a 7500 Fast Real-Time Cycler (Applied Biosystems, Foster City, CA, USA). Expression was normalized relative to levels of GAPDH or HPRT (as indicated in figures). Primer sequences are available on request.

### Statistical analysis

All statistical analyses were performed with Graphpad Prism 5.0 software. Statistical tests included: Shapiro-Wilk normality test, nonparametric Wilcoxon matched-pairs signed-rank test, and nonparametric Mann-Whitney test. P values less than 0.05 were considered significant. Because data sets were not normal (by the Shapiro-Wilk normality test), nonparametric tests were used instead of the parametric Student *t* tests. The Mann-Whitney test was used instead of the Student *t* test. The Wilcoxon matched-pairs signed-rank test was used instead of the paired Student *t* test.

## Additional Information

**How to cite this article**: Huynh, L. *et al*. Opposing regulation of the late phase TNF response by mTORC1-IL-10 signaling and hypoxia in human macrophages. *Sci. Rep.* **6**, 31959; doi: 10.1038/srep31959 (2016).

## Supplementary Material

Supplementary Information
