# Supplementary material for: Opposing regulation of the late phase TNF response by mTORC1-IL-10 signaling and hypoxia in human macrophages
Source: Sci Rep. 2016 Aug 25;6:31959. doi: 10.1038/srep31959 (PMC4997257; doi:10.1038/srep31959)
Supplement: Supplementary Information [file srep31959-s1.pdf]

Title: Opposing regulation of the late phase TNF response by mTORC1-IL-10 signaling and hypoxia in human macrophages

Authors: Linda Huynh, Anthony Kusnadi, Sung-ho Park, Koichi Murata, Kyung-Hyun Park-Min, and Lionel B. Ivashkiv

Supplementary Table SI. Genes induced with delayed and sustained kinetics after inflammatory activation of human macrophages.

Supplementary Table SII. Genes induced after 24 hr of TNF stimulation of human macrophages.

Supplementary Figure S1. TNF activates STAT3 with slower kinetics than does LPS in primary human macrophages.

Supplementary Figure S2. Culture in 1% oxygen induces expression of classic hypoxia-inducible genes and HIF-1α protein in primary human macrophages.

Supplementary Figure S3. Control TNF-inducible genes that are not regulated by hypoxia or mTOR.

Supplementary Figure S4. Immunoblot of proteins that are modulated by TNF and hypoxia.

Supplementary Figure S5. High level of purify of isolated $CD14^{+}$ cells from human PBMCs.

Supplementary Figure S6. Assessment of macrophage maturation markers after 48 hours *in vitro* culture.

Supplementary Table SI. Genes induced with delayed and sustained kinetics after inflammatory activation of human macrophages.

Fold Change	Common Name	Unstimulated		1 hour		3 hour		6 hour		24 hour	
19,265	CXCL13	3.305	A	16.76	A	8.945	A	1,227	P	21,482	P
3,143	NKG7	12.33	A	13.59	A	99.97	A	96.44	A	19,284	P
1,520	CCL23	12.2	A	30.64	A	113.2	P	1,412	P	13,822	P
1,026	SIGLEC10	116.8	A	175.5	A	103.8	A	169.2	P	21,198	P
959.5	CLU	10.54	A	10.83	A	5.211	A	16.81	A	11,775	P
751.1	TNIP3	4.026	A	604	P	27,847	P	21,453	P	4,074	P
658.9	ARNT2	6.779	A	18.19	A	96.6	P	515.4	P	2,302	P
523.3	C1S	6.212	A	5.866	A	3.57	A	164.5	P	5,435	P
407.7	TNFAIP6	120.2	P	4,853	P	29,478	P	33,118	P	31,923	P
380.2	FAM20A	4.087	A	5.301	A	90.53	A	1,193	P	4,850	P
378.8	NDP	25.38	A	31.11	A	184.7	P	2,772	P	7,481	P
315.9	IL7R	44.55	A	1,018	P	27,049	P	34,186	P	23,869	P
275.7	KIAA1199	2.233	A	4.059	A	458.5	P	5,003	P	3,585	P
270.8	RRAD	9.258	A	658.9	P	883.1	P	138.9	P	1,420	P
255.4	LAMP3	15.23	A	64.45	A	6,712	P	19,104	P	9,251	P
245.5	SLAMF1	9.055	A	50.1	A	10,401	P	19,659	P	4,479	P
231.4	IL2RA	6.359	A	64.97	A	988.6	P	8,690	P	5,546	P
182.5	CYP27B1	9.942	A	21.12	A	566.5	P	6,907	P	6,447	P
181.8	CSPG2	321.7	P	362.1	P	163.9	P	802.3	P	13,194	P
169.5	CXCL5	195.8	P	1,462	P	1,997	P	2,828	P	17,694	P
168.2	INDO	72.92	A	80.55	A	3,758	P	30,660	P	22,631	P
165	CCL15	3.717	A	11.4	A	318.9	P	2,309	P	1,627	P
163.6	MYH11	17.27	A	16.63	A	7.905	A	23.95	A	2,791	P
163.5	KCNJ15	49.05	P	66.5	M	69.47	P	330.8	P	4,147	P
142.1	IL1B	331.2	P	49,409	P	43,191	P	34,703	P	18,603	P
140.5	HS3ST3B1	81.75	P	359.9	P	4,979	P	17,271	P	14,283	P
135.2	RHOH	67.1	A	192	A	904.6	P	972.3	P	1,681	P
133.8	ENPP2	299.4	P	541.4	P	465.7	P	4,569	P	20,498	P

Supplementary Table SI continued.

Fold Change	Common Name	Unstimulated		1 hour		3 hour		6 hour		24 hour	
121.7	TM6SF1	55.04	M	51.35	A	74.34	P	173.1	P	481.2	P
111.4	LOC51334	23.24	A	12.9	A	69.52	P	368.9	P	882.7	P
108.4	FPRL1	91.24	P	111	A	272	P	1,037	P	3,530	P
107.5	CLECSF8	144.6	P	227.4	P	704.1	P	2,427	P	9,809	P
106.1	ORM2	3.649	A	4.201	A	6.853	A	26.53	A	682.6	P
104.9	SOCS3	125.8	P	7,248	P	18,565	P	9,571	P	4,558	P
100.2	MT1K	6.114	A	557.1	P	2,969	P	1,732	P	20,358	P
100	SLC8A3	19.04	A	14.92	A	35.37	A	330.3	P	688.4	P
99.65	CCL5	130.7	P	1,355	P	18,587	P	24,483	P	18,488	P
96.91	C1RL	87.1	P	101.5	P	29.74	A	355.9	P	5,665	P
93.22	LOC285758	7.004	A	13.93	A	45.3	A	284.6	P	683.8	P
90.47	PDCD1LG2	3.186	A	3.532	A	4.37	A	215.5	M	272.7	P
86.67	PTGES	21.03	A	105	A	35.13	A	132.4	A	1,511	P
83.77	ISG20	27.19	A	163	P	14,977	P	20,244	P	1,928	P
82.39	VILL	38.06	A	129.3	A	25.27	A	123.7	A	1,986	P
80.8	ACHE	7.116	A	10.48	A	34.95	A	27.3	A	753.3	P
79.06	CCL19	20.85	A	69.82	A	676.2	P	11,110	P	7,181	P
76.19	MAP1LC3A	231.8	P	271.7	P	621.4	P	1,922	P	15,554	P
75.93	BF	115.6	A	88.16	A	1,067	P	9,732	P	15,310	P
73.68	EBI3	10.98	A	71.64	A	2,415	P	7,249	P	6,717	P
72.43	SLC2A6	110.6	P	2,325	P	16,247	P	19,751	P	16,259	P
71.94	CAMK2A	6.311	A	11.79	A	27.03	A	162.5	A	621.5	P
71.8	MN1	115.7	M	238.3	A	692.6	P	1,725	P	3,528	P
71.11	FYN	60.54	A	61.44	A	328.8	P	201.4	P	1,360	P
69.93	SLC39A8	178.7	P	269.2	P	2,050	P	8,512	P	13,811	P
67.05	CLECSF9	578.9	P	1,298	P	2,374	P	4,842	P	18,916	P
66.33	HSD11B1	79.93	A	132	P	535.2	P	11,833	P	15,745	P
64.53	GJB2	22.47	A	1,457	P	3,886	P	7,655	P	5,736	P

Supplementary Table SI continued.

Fold Change	Common Name	Unstimulated		1 hour		3 hour		6 hour		24 hour	
61.64	T1A-2	79.52	A	86.68	A	107.7	M	680.1	P	3,575	P
61.64	KCNJ10	5.489	A	11.61	A	6.939	A	51.94	A	240.4	P
61.22	HAPLN3	68.24	A	164.1	A	416.3	P	1,156	P	1,555	P
61.16	S100A12	211	P	157.2	P	217.3	P	715.7	P	6,494	P
59.46	GCH1	379.7	P	5,331	P	30,974	P	30,598	P	15,577	P
58.07	CCR2	862	P	338.3	P	160.8	P	411.5	P	3,820	P
56.11	FPR1	458.7	P	319.4	P	393.9	P	1,179	P	15,564	P
55.42	DUSP5	84.89	P	4,634	P	10,906	P	3,672	P	2,168	P
54.46	MUC1	16.34	A	52.36	A	114.5	P	253.9	P	564.6	P
53.88	SLC1A2	14.25	A	29.18	A	110.4	P	147.8	P	708.8	P
51.74	NK4	5.787	A	6.165	A	44.25	A	342.9	A	1,747	P
50.74	TLE1	56.73	P	87.5	P	397	P	402.4	P	400.9	P
50.66	CCL20	8.233	A	28,112	P	33,932	P	21,422	P	1,046	P
49.77	FCAR	3.898	A	107.5	A	33.04	A	10.31	A	202.6	P
49.45	LOC118430	17.21	A	15.67	A	14.59	A	194.7	P	682.2	P
47.67	GPR84	241.5	P	2,380	P	3,759	P	9,038	P	10,228	P
46.85	IL1A	38.76	A	14,675	P	5,266	P	2,783	P	425.4	P
46.5	SLC8A1	132.7	A	112.5	A	41.03	A	19.83	A	687.3	P
45.75	AK3	10.75	A	8.224	A	1,069	P	1,952	P	565	P
45.38	SOCS2	13.35	A	45.83	A	558.8	P	308	P	123.9	P
44.37	MGC61633	70.93	A	11.35	A	23.74	A	48.12	A	749.9	P
44.08	CRABP1	14.38	A	11.37	A	108.6	A	9.149	A	268.6	P
43.42	MGC48332	95.1	A	104	A	220.1	P	586.7	P	565.5	P
43.1	CD44	24.99	A	326.3	P	648.5	P	503.6	P	198.2	P
42.87	PTPRF	55.55	A	15.09	A	37.72	A	76.03	A	431.5	P
42.66	AQP9	226.5	A	435.8	P	9,632	P	18,200	P	18,170	P
42.31	ASS	44.43	A	74	A	48.37	A	198	P	1,176	P
42.19	ARNTL2	55.11	A	10.72	A	75.93	A	537.3	P	1,193	P

Supplementary Table SI continued.

Fold Change	Common Name	Unstimulated		1 hour		3 hour		6 hour		24 hour	
41.82	EHD1	14.21	A	799.6	P	7,959	P	4,791	P	395.3	P
41.2	FCGR1A	28.93	A	40	A	31.5	A	85.2	A	188.8	P
40.99	ITGB8	0.733	A	20.12	A	427.3	P	1,738	P	783.9	P
39.69	RASGRP1	3.798	A	3.876	A	246.1	P	892.4	P	880.2	P
39.19	NNMT	27.3	A	25.62	A	14.06	A	133.7	A	578.8	P
37.83	ChGn	79.53	P	52.73	A	8.363	A	34.8	A	1,956	P
37.13	SLCO5A1	54.78	A	9.21	A	46.91	A	162.5	A	123.4	P
37.1	PDCD1LG1	81.71	A	188.6	P	19,535	P	21,239	P	4,189	P
37.02	OSM	183.3	P	2,301	P	245.2	P	505.7	P	498.8	P
36.82	G0S2	167.9	P	2,083	P	9,135	P	25,315	P	5,751	P
36.67	JAK3	239.3	P	263.2	P	585.6	P	2,604	P	7,903	P
36.67	BTBD11	36.57	P	60.36	P	22.62	A	9.431	A	91.44	P
36.6	MCOLN2	91.2	A	85.36	A	376.2	P	4,491	P	3,656	P
36.08	KIAA0984	28.13	A	47.47	A	150.2	P	136.1	P	489.8	P
34.71	SPTB	41.49	A	36.82	A	10.66	A	82.23	A	599.4	P
34.59	HEY1	15.13	A	1,329	P	551.8	P	1,171	P	441.5	P

Supplementary Table SI shows hybridization signals from a microarray experiment of a time course of inflammatory stimulation of primary human macrophages. The unstimulated condition (column 3) represents signals from cells at the beginning of the time course. Fold change values reflect the ratio of gene expression in stimulated cells at the 24 hour time point (column 7) versus unstimulated cells harvested at the same time (not shown). A (absent), P (present) and M (marginal) represent calls whether a gene is expressed relative to background made by Affymetrix software based on internal controls. One out of two similar experiments with an extensive time course of macrophages stimulated with fibrinogen, which induces autocrine TNF, is shown. An additional microarray experiment using macrophages directly stimulated with TNF for 24 hours showing induction of similar genes at the 24 hour time point is shown in Supplementary Table II. TNF-induced gene expression at the 24 hour time point was confirmed for > 20 genes in independent donors.

Supplementary Table SII. Genes induced after 24 hr of TNF stimulation of human macrophages.

Common Name	Fold Change	Common Name	Fold Change
TNFAIP6	525.53	CYP27B1	21.00
LAMP3	236.92	SLC2A6	19.93
IL1B	133.13	SLC1A2	19.03
PTGES	122.62	C1S	16.27
GJB2	111.45	ARNTL2	15.95
CLU	103.61	KIAA1199	15.64
CXCL5	88.05	RASGRP1	15.56
IL7R	82.10	MYH11	15.56
CCL5	76.34	MN1	14.92
MAP1LC3A	71.87	RRAD	13.98
SLC39A8	70.22	S100A12	12.88
HS3ST3B1	66.71	TLE1	11.54
ITGB8	55.83	CD44	10.51
SLAMF1	44.80	ACHE	10.46
CCL19	42.47	RHOH	10.08
IL2RA	41.50	KCNJ15	9.76
G0S2	40.29	SOCS3	8.94
SIGLEC10	36.11	HSD11B1	8.68
HEY1	35.30	FYN	8.40
CCL23	32.92	IL1A	8.25
MCOLN2	32.03	SOCS2	7.65
GPR84	28.64	ISG20	7.40
FCAR	27.72	AQP9	7.07
ARNT2	26.46	CCL20	6.43
TNIP3	25.09	VILL	6.43
DUSP5	24.80	EHD1	6.40
ENPP2	24.08	OSM	5.99
GCH1	21.76	KCNJ10	5.85
C1RL	5.64	CCR2	3.72
PDCD1LG2	5.40	SLCO5A1	3.63
FAM20A	4.18	JAK3	3.44
BTBD11	4.07	LOC285758	2.66
FPR1	4.06	SLC8A3	2.17
HAPLN3	4.00	PTPRF	2.12
SLC8A1	3.82		

Supplementary Table SII shows fold induction (columns 2, 4) of the genes in Supplemental Table I in a microarray analysis of primary human macrophages stimulated directly with TNF for 24 hr. TNF-induced gene expression at the 24 hour time point was confirmed for > 20 genes in independent donors.

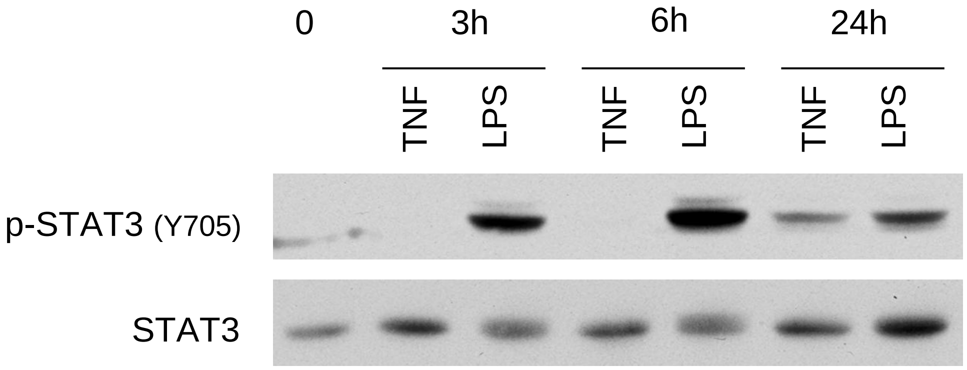

Supplementary Figure S1. TNF activates STAT3 with slower kinetics than does LPS in primary human macrophages. Primary human macrophages were stimulated for the indicated times with TNF or LPS (10 ng/mL). LPS activated STAT3 after 3 hours whereas TNF-induced activation of STAT3 was not apparent until 24 hours after stimulation. Data is representative of 2 donors.

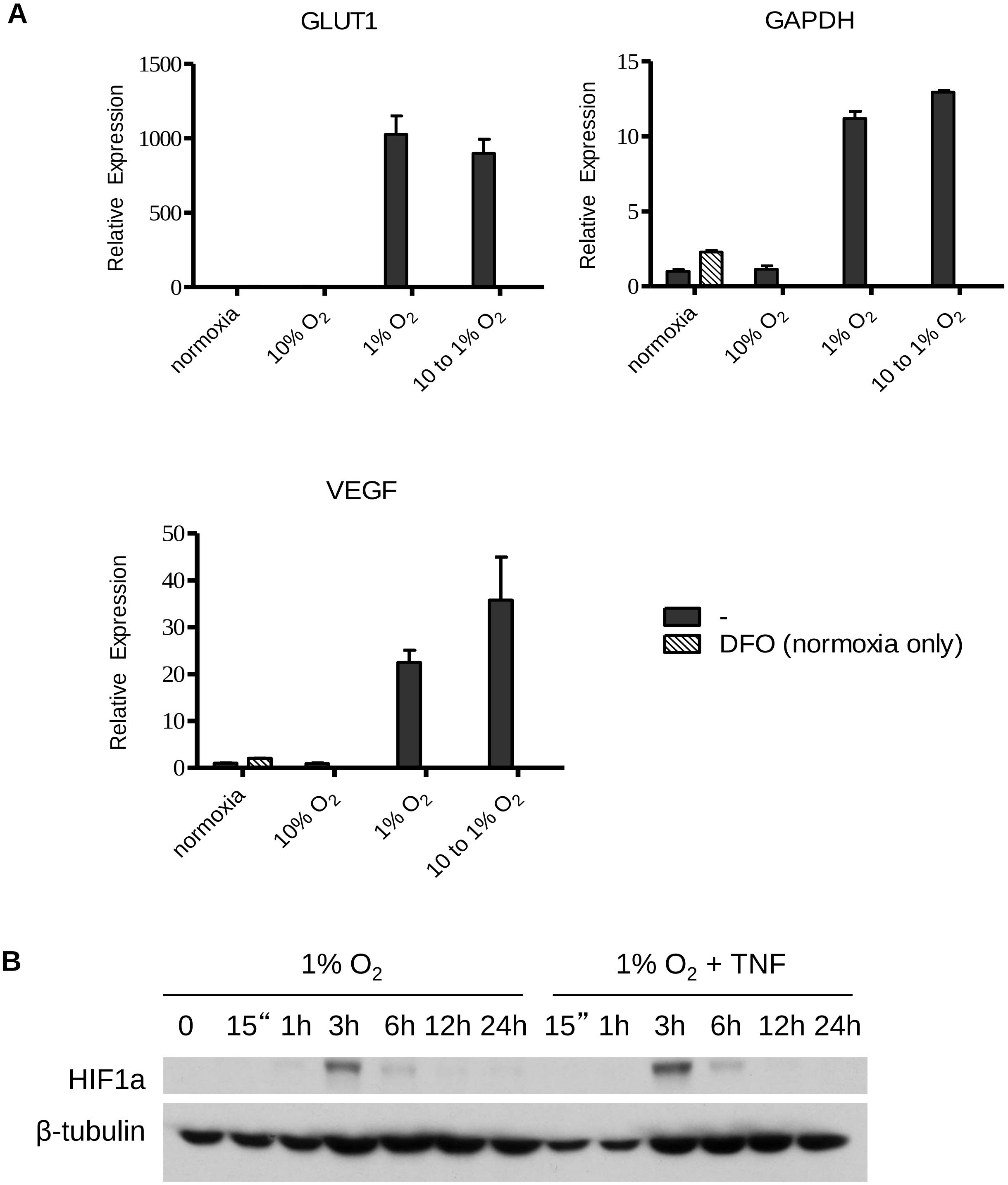

Supplementary Figure S2. Culture in 1% oxygen induces expression of classic hypoxia-inducible genes and HIF-1α protein in primary human macrophages. Primary human macrophages were incubated at normoxia (ambient 20% gas-phase) or in oxygen-sensor regulated incubators (1% or 10% gas phase) after differentiation with M-CSF at normoxia. “10 to 1%” refers to cells differentiated with M-CSF at 10% oxygen and then transferred to 1% conditions. At normoxia, the hypoxia mimetic desferoxamine (DFO) was also used. (A) Cells were incubated for 24 hours at the indicated oxygen concentrations. mRNA expression was measured by qPCR, and results are presented as mean ± SD of duplicate wells normalized relative to HPRT. Data are representative of at least 3 independent experiments. (B) Whole cell lysates were immunoblotted with Abs against HIF-1α and β-tubulin. Data are representative of 3 independent experiments.

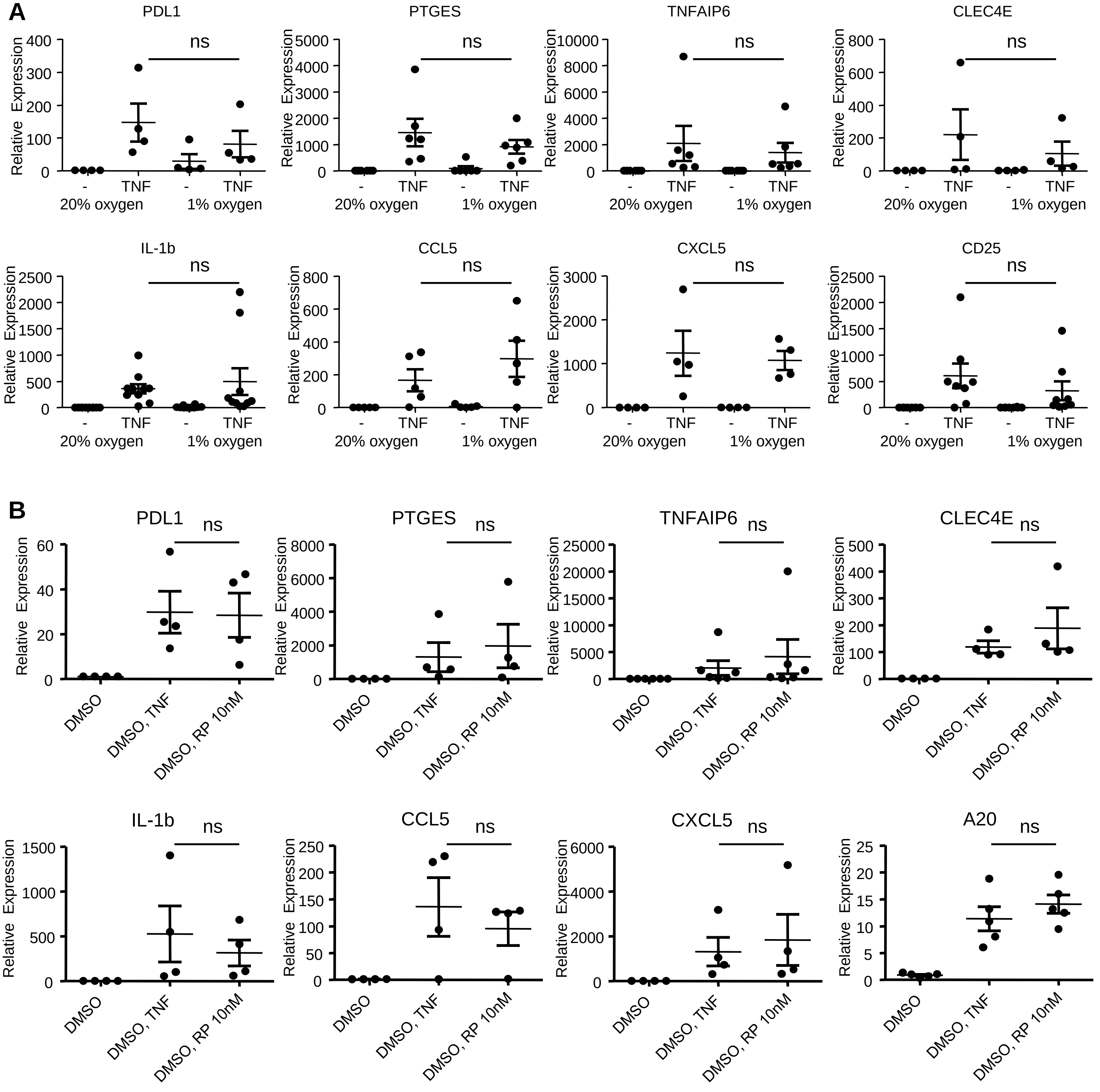

Supplementary Figure S3. Control TNF-inducible genes that are not regulated by hypoxia (A) or mTOR (B). (A) Primary human macrophages were differentiated with M-CSF at normoxia (20% O_2) and then moved into 1% O_2 for 3 h before TNF (10 ng/mL) was added for an additional 24 h. Control cells were maintained at normoxia. mRNA expression was measured by qPCR and normalized relative to HPRT. Data from at least 4 individual donors was analyzed using the Wilcoxon matched-pairs signed-rank test. (B) Primary human macrophages were pre-incubated for 1 hour with vehicle control DMSO or the mTOR inhibitor rapamycin (RP) and then stimulated with TNF (10 ng/mL) for 24 h. These experiments were performed at normoxia. mRNA expression was measured by qPCR and normalized relative to HPRT. Data from at least 4 individual donors are presented as mean ± SEM and was analyzed using the Wilcoxon matched-pairs signed-rank test.

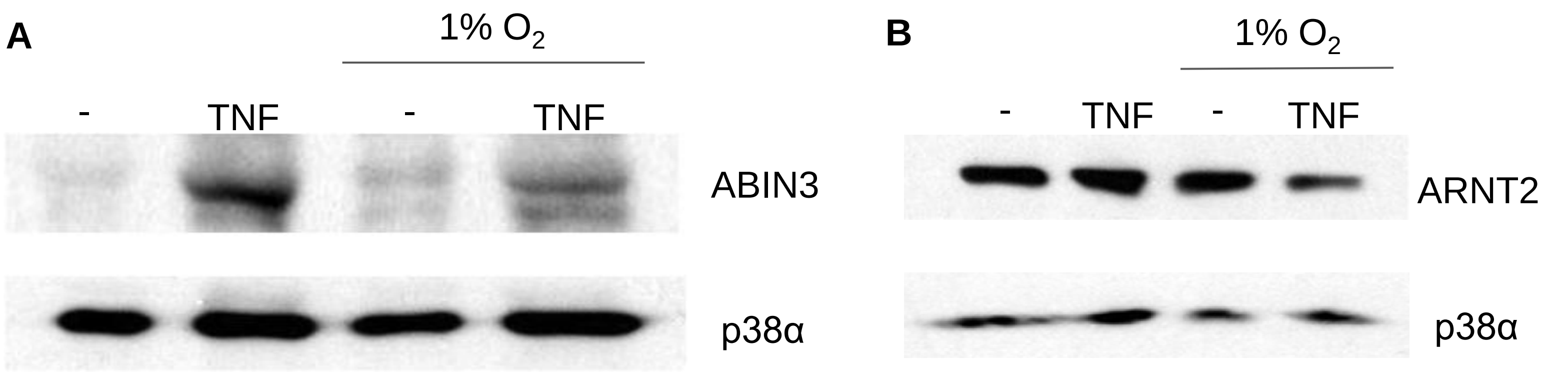

Supplementary Figure S4. Immunoblot analysis of proteins that are modulated by TNF and hypoxia. Cells were stimulated with TNF (10 ng/ml) for 24 hours in either normoxic or hypoxic conditions. After 24 hours, whole cell lysates were collected and immunoblotted with antibodies against (A) ABIN3, (B) ARNT2. p38α was used as loading control. Data is representative of 3 donors.

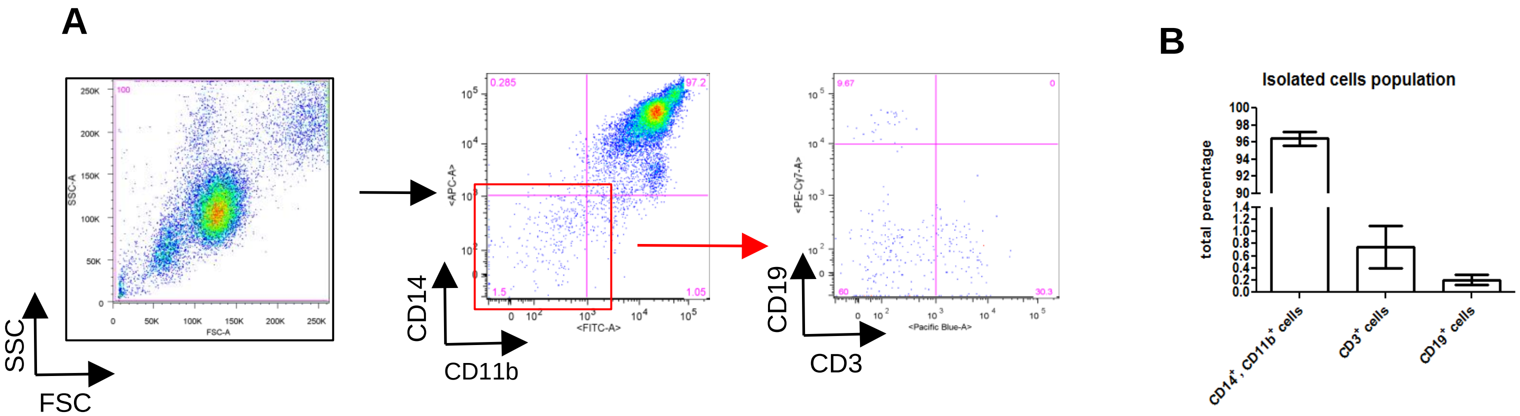

Supplementary Figure S5. Flow cytometric characterization of CD14^{+} cells isolated from human PBMCs. (A) Isolated CD14^{+} cells were stained with antibodies against CD14, CD11b, CD3 and CD19. The second panel shows two color analysis of CD11b and CD14. The double negative cell population was gated on (square in center panel) and analyzed for expression of CD3 (T cells) and CD19 (B cells) (right panel). (B) Mean percentage of CD14+CD11b+, CD3+ and CD19+ cells in isolated cells from 3 donors.

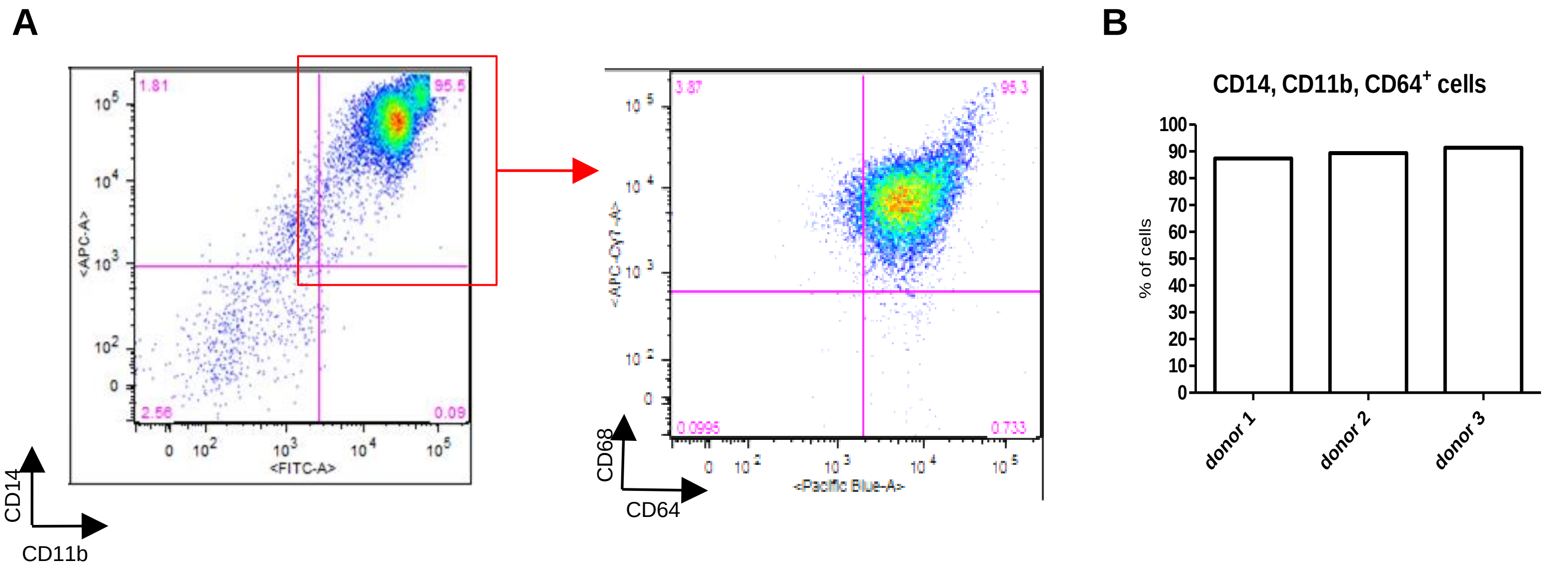

Supplementary Figure S6. Assessment of macrophage markers after 48 hours *in vitro* culture. Isolated CD14-positive monocytes were cultured *in vitro* for 48 hours in the presence of M-CSF (10ng/ml). After 48 hours of culture, macrophage markers were examined using FACS. Cells were stained with antibodies against CD14, CD11b, CD64 and CD68. (A) Cells that were double positive for CD14 and CD11b were gated on (square in left panel), were analyzed for CD64 and CD68 expression (right panel). Data is representative of 3 donors. (B) Mean percentage of $CD14^{+}CD11b^{+}CD64^{+}$ cells in 3 experiments with different donors.
